# Exploring the chemical space of protein–protein interaction inhibitors through machine learning

**DOI:** 10.1038/s41598-021-92825-5

**Published:** 2021-06-28

**Authors:** Jiwon Choi, Jun Seop Yun, Hyeeun Song, Nam Hee Kim, Hyun Sil Kim, Jong In Yook

**Affiliations:** 1 Department of Oral Pathology, Oral Cancer Research Institute, Yonsei University College of Dentistry, Seoul, South Korea; 2 Met Life Sciences Co., Ltd., Seoul, South Korea

**Keywords:** Computational biology and bioinformatics, Drug discovery

## Abstract

Although protein–protein interactions (PPIs) have emerged as the basis of potential new therapeutic approaches, targeting intracellular PPIs with small molecule inhibitors is conventionally considered highly challenging. Driven by increasing research efforts, success rates have increased significantly in recent years. In this study, we analyze the physicochemical properties of 9351 non-redundant inhibitors present in the iPPI-DB and TIMBAL databases to define a computational model for active compounds acting against PPI targets. Principle component analysis (PCA) and *k*-means clustering were used to identify plausible PPI targets in regions of interest in the active group in the chemical space between active and inactive iPPI compounds. Notably, the uniquely defined active group exhibited distinct differences in activity compared with other active compounds. These results demonstrate that active compounds with regions of interest in the chemical space may be expected to provide insights into potential PPI inhibitors for particular protein targets.

## Introduction

Protein–protein interactions (PPIs) play central roles in almost all intracellular and extracellular biological processes and are essential to the mechanisms of various diseases and pathological conditions such as neurodegeneration, cardiovascular diseases, and cancer^1–4^. Approximately 650,000 PPIs are known to be present in each human cell, suggesting that 650,000 potential targets may exist for modifying cellular biological functions using drugs^5–8^. Although PPI-related crucial functions have been demonstrated in numerous disease states and have attracted increasing research attention as an emerging class of molecular targets, they have been conventionally considered intractable for small-molecule modulators owing to druggability issues and their large and flat interfaces^9–11^. However, small-molecule PPI inhibitors and PPI-focused chemical libraries have recently been improved as a result of the development of high-throughput experiments and in silico technologies such as cheminformatics and machine learning tools^12–19^. Historically, the molecular topography of most known PPI inhibitors has been shown to share common surface features such as being more shallow, large, and hydrophobic than typical orally available drugs. Thus, PPI inhibitors are larger, more hydrophobic, more rigid, and contain multiple aromatic rings^5,8^. Over recent decades, many PPI inhibitors have been screened for their action against PPI-related targets and have achieved considerable clinical success in the treatment of autoimmune diseases and cancer^20,21^. Moreover, the physicochemical and pocket properties of PPI inhibitors have been identified through PPI-specific database analysis; studies have suggested that PPI target classes with matching regions in both chemical and target spaces could facilitate the development of iPPIs to the stage of drug candidates^1,22,23^.

However, individual PPI target analyses based on PPI-specific databases incorporating the chemical and physical characteristics of these compounds have thus far remained insufficient. Moreover, no study has focused on the methods by which knowledge of an “active” or “inactive” result from a bioassay could be used to design PPI inhibitors; successful examples have also not been reported in the relevant literature. Compared with most non-PPI inhibitors, the average molecular weight of PPI inhibitors is significantly greater than 500 Da; however, this trend has been driven, in large part, by the contribution of peptide-based compounds^2,24^. Therefore, further analysis must be conducted based on a limited region of small molecules in the PPI database, referred to as the biologically relevant chemical space for each PPI target.

In this study, we compared active and inactive datasets to identify promising active compounds for each PPI target. To characterize chemicals and predict their experimental activities, cheminformatics techniques with very high reliability are required to enable the evaluation of experimental values such as pKi or IC50. Thus, we developed predictive computational models to identify and prioritize the most promising chemicals. In addition to investigating the physicochemical property distributions of these compounds using molecular descriptors, we also visualized them in structural chemistry space using principal component analysis (PCA) and a *k*-means clustering algorithm. Then, we determined PPI-specific targets with regions of interest of the active group in the chemical space and observed that active compounds in such regions of interest exhibited potent profiles compared to weakly active and inactive compounds. We also show that the seven molecular descriptors used as the basis of the computational model can provide useful information regarding the unique chemical characteristics of Bcl-2 active compounds and assist in differentiating most active Bcl-2 inhibitors^25–28^.

## Materials and methods

### Dataset preparation

The iPPIs dataset was generated from iPPI-DB (https://ippidb.pasteur.fr/) and TIMBAL (http://mordred.bioc.cam.ac.uk/timbal/) databases^15,16,19^. After removing redundant compounds, non-redundant datasets comprising 1756 and 7610 compounds were obtained for the iPPI-DB and TIMBAL databases, respectively. The activity values (K_d_, Ki, IC_50_, and EC_50_) of the various compounds were used to classify them as active or inactive. Compounds with activity values of less than 30 μΜ were categorized as “active” compounds. The dataset used for PCA and clustering was constructed using 9351 iPPI compounds. The two datasets (iPPI-DB dataset and active portion of TIMBAL dataset) were merged, 15 duplicated compounds were removed. Subsequently, we constructed datasets for each PPI target, and sets of Bcl-2 and MDM2 protein compounds were obtained with 992 and 932 members, respectively, and subjected to further analysis.

### Principal component analysis (PCA) and cluster analysis

A molecular descriptor is defined as a numerical description computationally representing physical and chemical information of compounds. Such descriptor parameters were generated for the two datasets using the molecular descriptor calculator included in the QikProp module of the Schrödinger platform (Maestro, Schrödinger, LLC, New York, NY, 2020). The calculated descriptors included molecular weight, number of hydrogen bond acceptors, number of hydrogen bond donors, ALogP, number of rotatable bonds, number of aromatic rings, and polar surface area. PCA is a multivariate statistical method used in exploratory data analysis. It allows the representation of the property space by perspective projection into a principal component plane (PC1, PC2), encoding the data from a given mathematical viewpoint^29–35^. To extract the most important information from the dataset, PCA was employed to explore the chemical space of iPPI inhibitors as a function of these seven molecular descriptors using the FactoMineR R package^36,37^. PCA-clustering values were calculated on the iPPI dataset using the *k*-means clustering method within the Factoextra R packages. All histograms and scatter plots were generated using the R software.

### Docking simulation

To understand the affinity of protein–ligand binding, a molecular docking approach was employed. Sets of 992 active Bcl-2 inhibitors were simulated by a docking program. To evaluate the binding mode and affinity of the dataset to the Bcl-2 target proteins, the crystal structures were retrieved from the RCSB Protein Data Bank (PDB ID:2YXJ). Molecular docking studies were performed using Glide (Schrödinger, LLC, New York, NY, 2020), which uses the OPLS-2005 force field, and refinement was performed according to the recommendations of the Protein Preparation Wizard^38,39^. LigPrep (Schrödinger, LLC, New York, NY, 2020) was used to generate the 3D structures of the ligands. The active grid was generated using the Receptor grid application in the Glide module. On a defined receptor grid, flexible docking was performed using the standard docking precision (SP) mode of Glide. The best docking pose for a given compound was selected based on the best scoring conformations from the Glide score.

## Results and discussion

### Development of iPPI datasets

To analyze the unique chemical properties of PPI inhibitors, we generated datasets of PPI inhibitors from the iPPI-DB and TIMBAL databases including small molecules inhibiting PPIs^15,16,19^. The datasets were checked for redundancy. The datasets contained 9351 non-redundant inhibitors. The iPPI-DB database contained 1756 inhibitors for 18 targets, while TIMBAL database contained 7610 inhibitors for 34 targets (Supplementary Fig. S1). We then classified the PPI inhibitor compounds present in the iPPI-DB and TIMBAL databases into active and inactive categories; the compounds with dose response values equal to or lower than 30 μM were retained as the active group, and others were classified as the inactive group.

**Figure 1:**
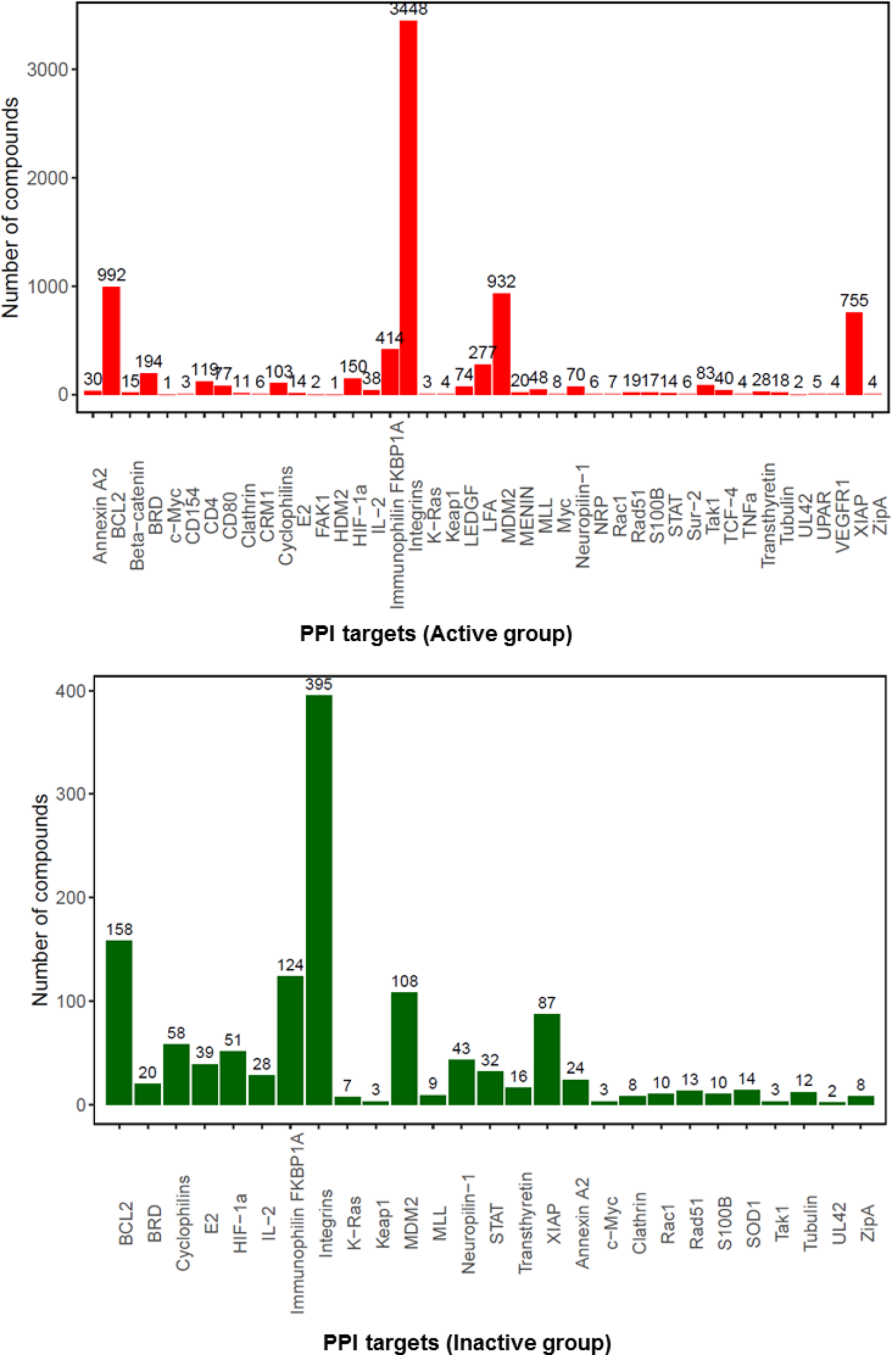
Distributions of compounds for target proteins of iPPI datasets. The colored histogram shows the frequency distribution against numbers of known compounds for each PPI target. Panels (**A**) and (**B**) indicate results for the active and inactive groups in the iPPI datasets, respectively.

Thus, the datasets containing the 9351 chemicals were designated as active iPPI datasets and inactive iPPI datasets; 8066 compounds were active (PPI inhibitors) and 1285 were inactive (non-PPI inhibitors). The active and inactive datasets were designed to compare the physicochemical properties of active and inactive compounds on PPI targets. As shown in Fig. 1, we observed that the frequency distribution of PPI inhibitors in the iPPI-DB and TIMBAL databases was biased to only a small number of targets. These results indicated that random sampling over entire PPI targets might cover only a limited number of compounds and targets. Analyzing each PPI target was expected to provide a more accurate result for the active dataset, which is conventionally performed for broad datasets of PPI inhibitors. Therefore, the selected iPPI datasets based on partially limited targets can be expected to provide some clues to establish key points regarding the differences between active and inactive compounds on each PPI target.

### Comparative study on molecular descriptors of iPPI datasets

**Figure 2:**
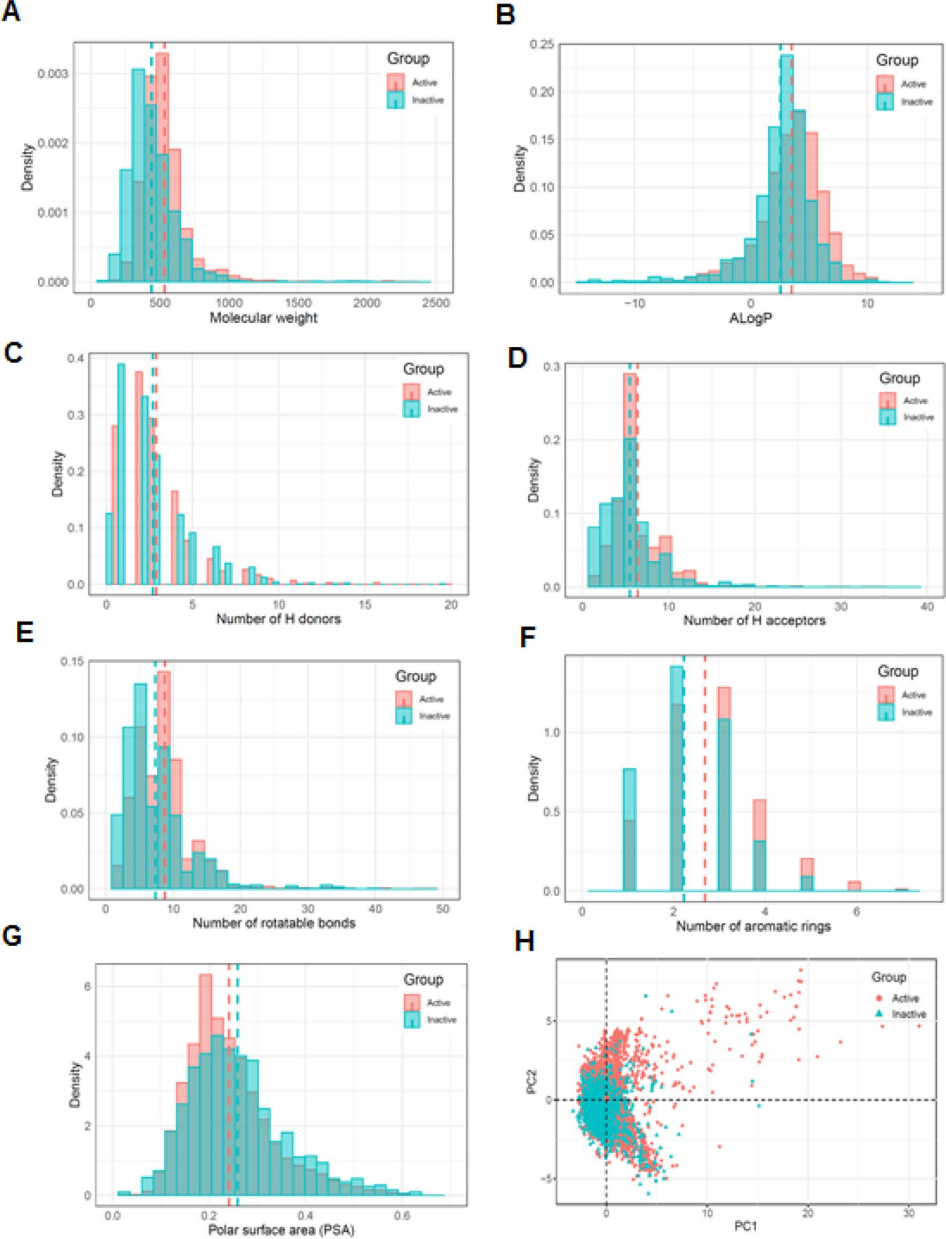
Physicochemical profile of compounds from iPPI datasets. (**A**–**G**) Chemical properties of the compounds from the iPPI datasets are compared using the histogram for the seven molecular descriptors. The dotted lines represent mean values, and the histogram bars of the active and inactive group are colored red and light green, respectively, whereas the dark green bar represents the overlap region. (**H**) Distribution of the chemical space of the compounds in the iPPI datasets according to principal component analysis. All histograms and scatter plots were generated using the R software.

To investigate the distinct features of the active and inactive datasets for iPPI compounds and to compare them, seven molecular descriptors of each molecule (molecular weight, ALogP, number of hydrogen bond acceptors, number of hydrogen bond donors, the number of rotatable bonds, number of aromatic rings, and polar surface area) were calculated and applied to perform PCA. The frequency distribution of physicochemical properties for the total iPPI datasets along with the active/inactive datasets is shown in Fig. 2, and a summary of the mean values of the seven molecular descriptors for dataset of each PPI target is given in Table 1.

**Table 1:** Summary of the chemical properties distributions and comparison of the active/inactive dataset.

Dataset	Size	MW (Da)	ALogP	HBA	HBD	NRB	NAR	PSA
Active	8066	533.4	3.51	6.00	2.89	8.78	2.69	0.24
Inactive	1285	438.5	2.53	5.51	2.69	7.37	2.22	0.26

*MW* mean of molar weight, *ALog P* mean of logarithm of the calculated octan-1-ol–water partition coefficient, *HBA* mean number of hydrogen bond acceptors, *HBD* mean number of hydrogen bond donors, *NRB* mean number of rotatable bonds, *PSA* mean number of polar surface area.

The histogram plots of the active/inactive dataset showed that the distributions of the seven molecular descriptors broadly overlapped between the two datasets (dark green regions shown in Fig. 2A–G). Comparison was performed using a two-sided Student’s *t-*test to determine whether the difference in descriptor distributions between the two datasets was significant, and the analysis showed that all descriptors were significantly different (p < 0.05). The mean values of the seven descriptors of the active dataset were greater than those of the inactive dataset (Table 1). By contrast, the mean value of the polar surface area (PSA) of the inactive dataset was significantly smaller than that of the active dataset. These results indicate that compounds within the active dataset possessed less polar surface area and were more lipophilic than those in the inactive dataset.

We performed PCA to compare the diversity of the two datasets. This projection provides a simplified view of the chemical space by reducing the dimensionality of the calculated descriptors according to a linear transformation. The accumulated variance showed that the two principal components, PC1 and PC2, represented 80.2% of the total variance, contributing 48.7% and 31.5%, respectively. As shown in Fig. 2H, PCA was applied to the seven descriptors, and the two datasets shared a common chemical space region. For the inactive dataset, the scatter plots of PC1 and PC2 showed that the chemical space was near that of the active dataset and largely clustered into a single group. However, we also identified that compounds in the active dataset occupied some distinct regions of interest. Therefore, separate datasets were constructed to characterize the distinct features of the chemical spaces of the active and inactive datasets.

### Diversity analysis for each PPI target dataset

To test whether the overlapping patterns in the chemical space between the two datasets we identified in Fig. 2H were statistically common key sources across the 43 PPI targets, we performed PCA on the active and inactive groups for each PPI target class. As shown in Supplementary Table S1, eight target proteins, including Bcl-2, BRD, Cyclophilins, HIF1a, ImmunophilinFKBP1A, Integrins, MDM2, and XIAP, were selected as containing more than 100 compounds in the active group and also being present in the inactive group.

**Figure 3:**
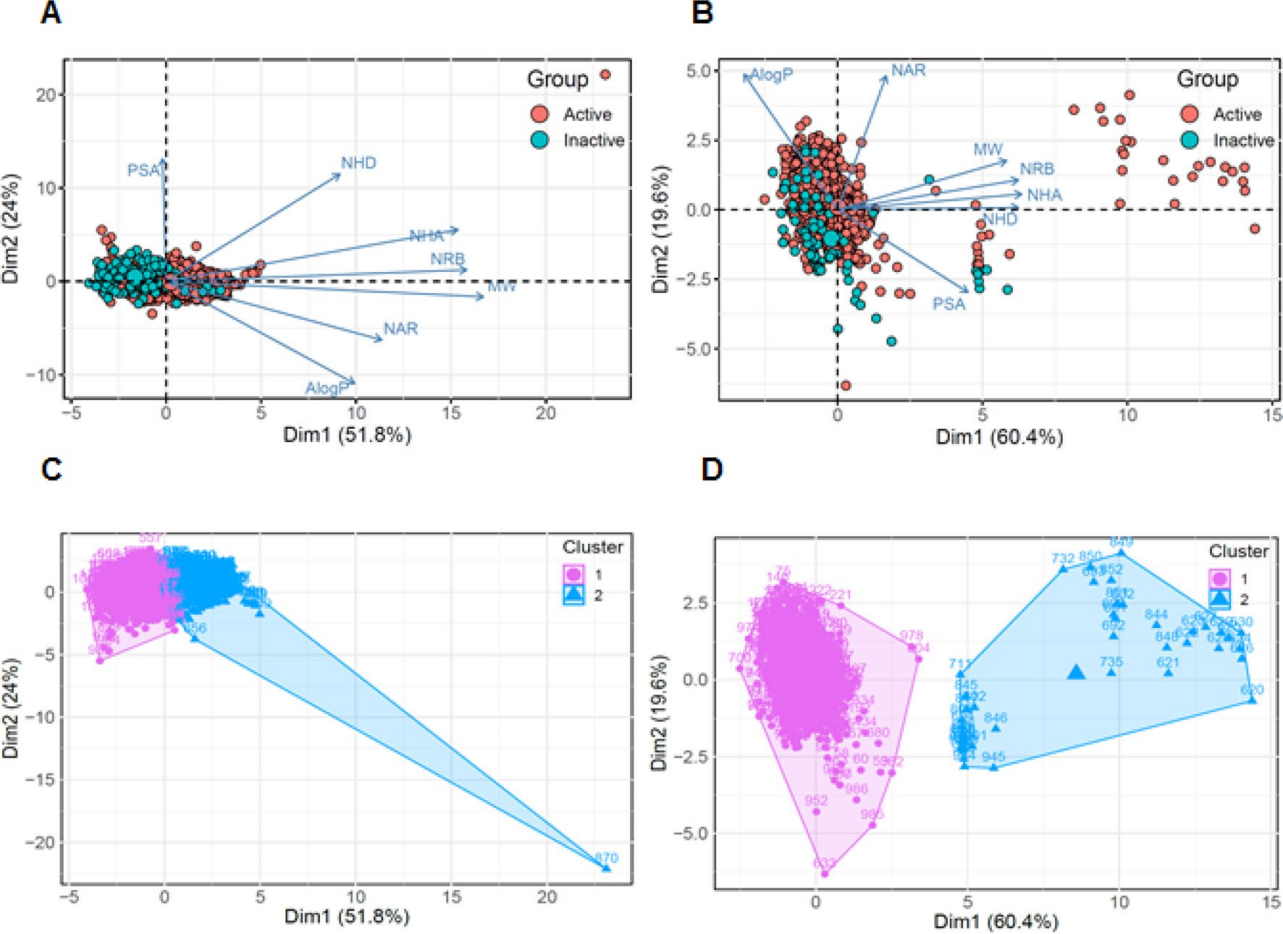
Visual representation of the chemical space of Bcl-2 and MDM2 dataset. Principal component analysis (PCA)-based clustering representing the comparison of the chemical space on active/inactive datasets in the Bcl-2 and MDM2 datasets. (**A**,**B**) Distribution of the chemical space of the compounds in the Bcl-2 and MDM2 dataset according to principal component analysis. The loading plot vectors are represented by arrows for each physicochemical property. (**C**,**D**) Data points are color-coded by cluster of molecules. The magenta and blue dots correspond to active compounds in the clusters 1 and 2, respectively.

The PCA scatter plot of the calculated physicochemical properties of each of the eight target proteins visually represents the active/inactive datasets, as described by the first two significant principal components (Fig. 3 and Supplementary Fig. S2). From the PCA of the eight target proteins, we identified that most of the target proteins shared a chemical space between the active and inactive groups.

In only the Bcl-2 and MDM2 datasets, the most prominent pattern observed in the chemical space was similar to that of the total PPI datasets, with both vast chemical diversity and regions of interest in the active group (Fig. 3A,B). As shown in Figure 3, the large number of data points in the active group for the Bcl-2 target protein differing significantly from the observations of the inactive group indicates a wider sampling distribution of the chemical space compared with the inactive dataset. The chemical spaces of the integrins and XIAP target proteins also showed a similar pattern of regions of interest to that of the total PPI dataset, but did not show a large number of data points with non-overlapping regions in the active group compared to the Bcl-2 and MDM2 datasets and were excluded from further study (Supplementary Fig. S2E,F).

### PCA-based clustering of Bcl-2 and MDM2 datasets

By performing PCA on the active/inactive dataset of Bcl-2 and MDM2, we identified similar patterns in the chemical space when PCA was applied to the complete dataset for all PPI targets. Particularly, the PCA-based visualization of the chemical space for the Bcl-2 and MDM2 inhibitors identified that a difference in active and inactive datasets was apparent. The clear difference in chemical space between the active and inactive dataset could aid in discriminating the most active compounds from moderate active and inactive compounds.

Next, we used k-means clustering to further investigate this idea and clustered the Bcl-2 and MDM2 datasets into seven molecular descriptor spaces (Fig. 3C,D). For the Bcl-2 dataset, we found the optimal clustering to be two clusters grouped together with active and inactive compounds, and that regions of interest were predominantly occupied by some active compounds in Cluster 2. In addition, through total iPPI dataset analysis, we confirmed that most active compounds present in the regions of interest belonged to the two target proteins Bcl-2 and MDM2 (Supplementary Fig. S3A,B). These results indicate that physicochemical descriptor-based clustering could accurately classify active and inactive compounds in the Bcl-2 dataset, such that compounds with similar activity were clustered together in the chemical space. In contrast, clustering using MDM2 datasets did not provide the optimum number of clusters grouped into active and inactive compounds in the chemical space. Figure 3D shows that the model did not accurately determine the classes of active and inactive compounds in MDM2 using the *k*-mean based clustering algorithm.

### Classification of physicochemical environment for Bcl-2 dataset

**Figure 4:**
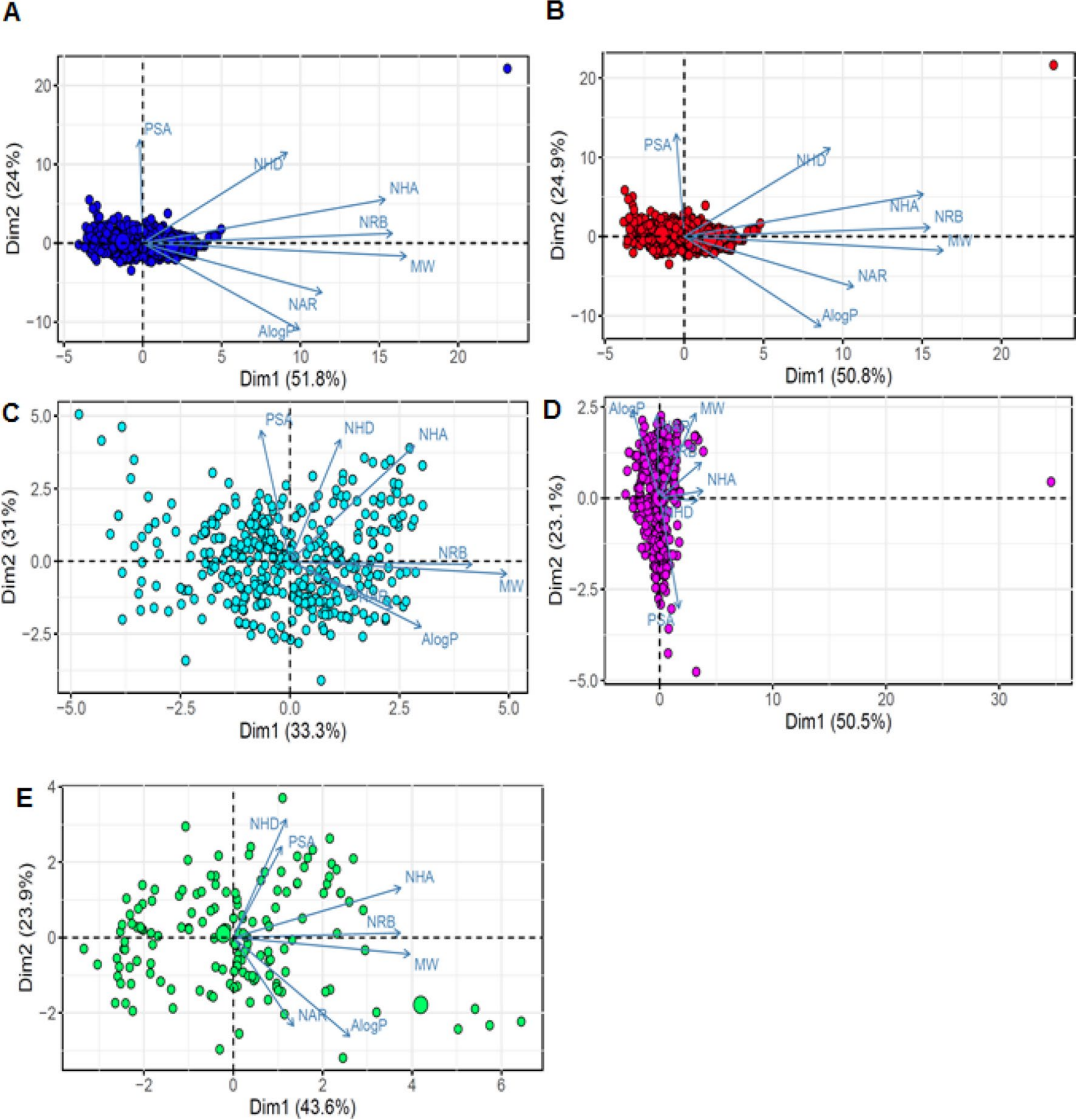
PCA plot of BCL-2 dataset. The visual representation was generated with principal component analysis of seven drug-like physicochemical properties. The loading plot vectors are represented by arrows for each physicochemical property. The blue dots (**A**), red dots (**B**), cyan dots (**C**), magenta dots (**D**), and green dots (**E**) represent Class 1, 2, 3, 4, and 5, respectively.

PCA and clustering of these seven chemical descriptors allowed us to establish a trend in the availability and distribution of the compounds, yielding five classes of physicochemical environments: Class 1, indicating total Bcl-2 compounds in Clusters 1 and 2; Class 2, indicating active compounds in Clusters 1 and 2; Class 3, indicating active compounds in Cluster 1; Class 4, containing active compounds in Cluster 2; and Class 5, representing inactive compounds in Cluster 1. Classes 1, 2, 3, 4, and 5 contained 1150, 992, 531, 461, and 158 Bcl-2 compounds, respectively (Fig. 4A–E). Interestingly, the PCA plot of active compounds in Cluster 2 (Class 4) showed a clear separation of active compounds in Cluster 1 (Class 3) (Fig. 3A). To identify new and unique characteristics of Bcl-2 active compounds present in Cluster 2, PCA was performed on the Bcl-2 datasets using the same seven molecular descriptors. As for Class 4, PC1 afforded the highest variance with a value of 50.5%, and PC2 provided the second-highest variance with a value of 23.1%. In particular, the loading plot shows that the positive end of PC1 was dominated by number of hydrogen bond acceptors (NHA), number of rotatable bonds (NRB), and number of hydrogen bond donors (NHD), thereby suggesting the importance of these descriptors in accounting for the variance of PC1 and PC2.

In contrast to that of Class 4, the loading plot of all other classes indicated that the positive end of PC1 was dominated by molecular weight, NRB, and NHA. Supplementary Figure S4 shows the top variables contributing to PC1 in a bar plot. The red dashed line on the graph indicates the expected average contribution. Therefore, the NHD descriptor, found only in Class 4, could be considered as an important contributor to the component. From these results, we identified a subset of descriptors having a clear difference in the profiles of active and inactive compounds among the Bcl-2 inhibitors. Taken together, a subset of descriptors comprising the NHD descriptor, appearing only in Class 4, was identified to characterize the main features of highly active compounds (Fig. 4D). These results could also be of benefit in comprehensively describing relevant physicochemical features required in the design of Bcl-2 inhibitors.

### Analysis of highly active group on Bcl-2 dataset

**Figure 5:**
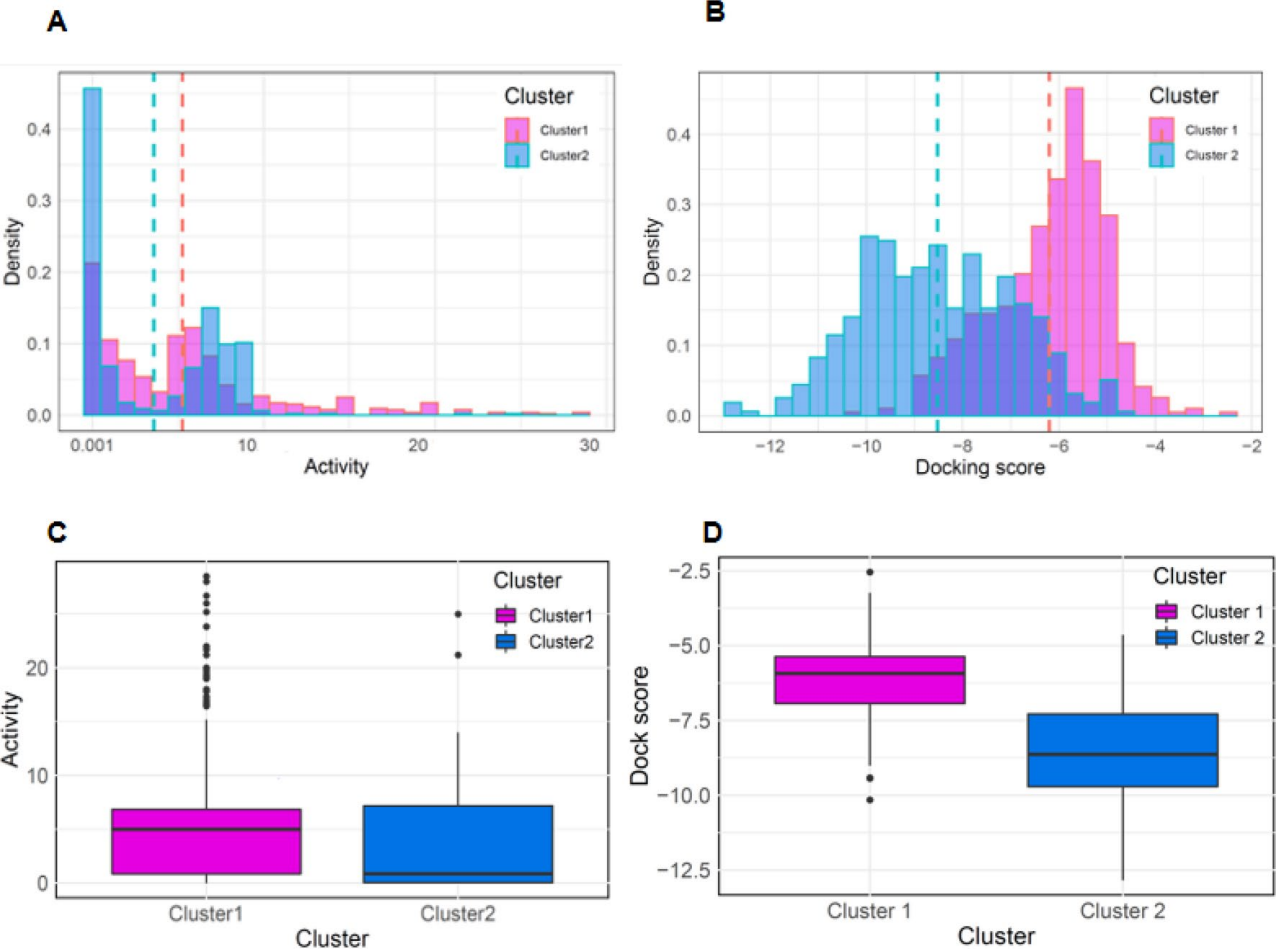
Distribution of active values and Glide scores of Cluster 1 and 2 active datasets. Histograms for (**A**) active values, (**B**) SP GlideScore distributions. The dotted lines represent mean values and the histogram bar of the Cluster 1 active set, and the Cluster 2 active set are colored magenta and blue, respectively, whereas the dark blue represents their overlap region. Boxplots for (**C**) activity values, (**D**) SP GlideScore distributions of active dataset for each the Cluster 1 and 2. The magenta and blue colors correspond to the active sets of Clusters 1 and 2, respectively.

To investigate the profile of active Bcl-2 compounds on Cluster 2, experimental active values and docking scores for the Cluster 1 and 2 active datasets were compared. Figure 5A,B show the distribution of the docking scores and the experimental active values for the Cluster 1 and 2 active datasets. The distribution of active values and docking scores for the Cluster 2 active dataset was also shifted left relative to that of the Cluster 1 active dataset. For active compounds in the Cluster 1 and 2 datasets, the mean activity values were 5.27 μM and 3.58 μM, respectively (Fig. 5C). The mean docking scores of active compounds in Clusters 1 and 2 were − 6.197 and − 8.524, respectively, indicating that the active compounds of Cluster 2 could be predicted to have higher binding affinity than the active compounds of Cluster 1 (Fig. 5D). These results indicate that the active compounds in Cluster 2 were more active on average. We showed that the seven molecular descriptors used in this study could discriminate highly active Bcl-2 compounds from Bcl-2 active compounds and provide valuable guidance for the design and discovery of potent Bcl-2 inhibitors.

## Conclusions

PPIs are essential to almost every cellular process from cell proliferation to cell metabolism; therefore, understanding PPIs is important in terms of human diseases. Recently, many PPI inhibitors have been widely studied as targets for PPI-inhibiting small ligands, and a large amount of data has been rapidly deposited in many public PPI databases. Nearly 650,000 PPIs have been identified in humans, and PPI-inhibiting drugs have been identified as a highly promising therapeutic approach. Nonetheless, PPIs have historically been considered difficult targets to modulate by small molecules because of the 3D structural and biophysical complexity of their interfacial features. Therefore, there is a need to identify chemical properties of PPI inhibitors based on curated high-quality PPI data. Exploration of the chemical space available in many public PPI databases provides an opportunity to analyze the molecular factors relevant to the bioactivity of PPIs.

The iPPI-DB and TIMBAL databases specialize in drug target PPIs and primarily focus on the chemical properties and information on the biological function of PPI-inhibiting ligands. Analysis of the properties of the two databases can provide a synergistic capability to identify novel chemicals inhibiting target PPIs. We hypothesized that chemical descriptors could be utilized to better characterize PPI inhibitors and thus construct target-specific models with enhanced prediction performance. Therefore, we used a *k*-means clustering algorithm in a PCA-based chemical space to analyze large datasets of PPI inhibitors to explore the biologically relevant chemical space. The PCA results showed that most of the target proteins shared a common chemical space between the active and inactive compound groups. However, only datasets of Bcl-2 and MDM2 among the eight target proteins showed an overlap pattern in chemical space similar to that of the total PPI datasets and had regions of interest in the active group. The selected machine learning techniques used in the present work could be successfully applied to confirm highly active compounds by evaluating the chemical properties of each PPI target. We compared the coverage of biologically relevant chemical spaces using active compounds of Bcl-2 targets to reveal regions populated by highly active compounds, referred to here as regions of interest.

Based on this analysis, we proposed a unique region in the chemical space that could be used to identify highly active compounds in the PPI-specific database. In addition, we explored Bcl-2 active compounds to define the binding affinity of various compounds against Bcl-2 targets using molecular docking simulation and showed that the compounds classified here as highly active compounds had higher binding affinity on average. These techniques could be successfully applied to define a novel Bcl-2 inhibitor profile on the PPI databases. Our analysis offers a comprehensive overview of Bcl-2 active compounds and should aid in the design of PPI-target-specific chemical libraries and the identification of potential active compounds for drug discovery.

## Supplementary Information

Supplementary Information.
