# Supplementary material for: Exploring the chemical space of protein–protein interaction inhibitors through machine learning
Source: Sci Rep. 2021 Jun 28;11:13369. doi: 10.1038/s41598-021-92825-5 (PMC8238997; doi:10.1038/s41598-021-92825-5)
Supplement: Supplementary file 1 — Supplementary Information. [file 41598_2021_92825_MOESM1_ESM.docx]

**Exploring the chemical space of protein–protein interaction inhibitors through machine learning**

Jiwon Choi^1, 2*^, Jun Seop Yun^1^, Hyeeun Song^1^, Nam Hee Kim^1^, Hyun Sil Kim^1^, Jong In Yook ^1, 2*^

^1^ Department of Oral Pathology, Oral Cancer Research Institute, Yonsei University College of Dentistry, Seoul, Korea

^2^ Met Life Sciences Co., Ltd., Seoul, Korea

^*^ Corresponding authors.

Jong In Yook*,* *E-mail*: [jiyook@yuhs.ac](mailto:jiyook@yuhs.ac)

Jiwon Choi*,* *E-mail*: [edccjw@gmail.com](mailto:edccjw@gmail.com)

Jiwon Choi and Jun Seop Yun contributed equally to this work

Supplementary Materials


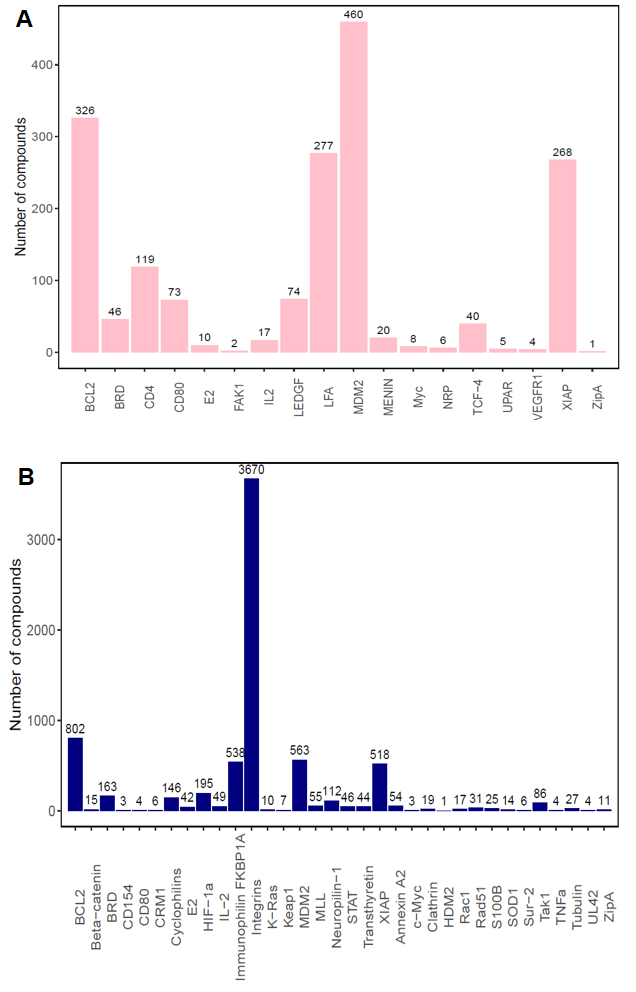


**Figure S1**. **Distributions of the compounds for target proteins of the commercial iPPI database.** The frequency distribution histograms are plotted against numbers of known compounds for each PPI target. Left and right panels indicate results for iPPI-DB and TIMBAL databases, respectively.


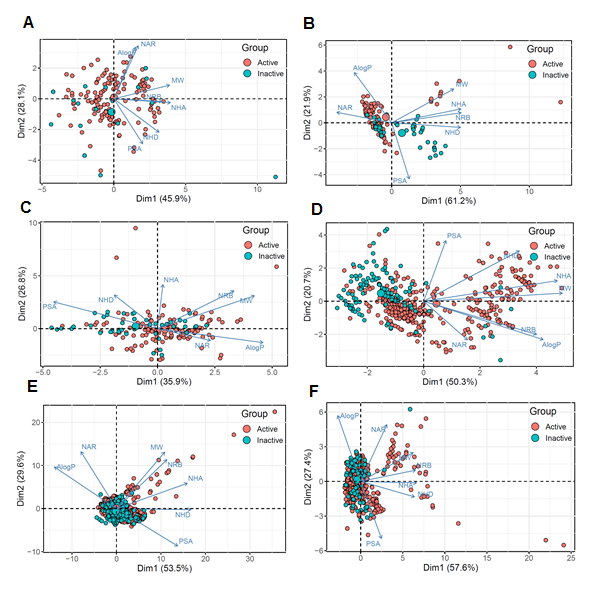


**Figure S2**. Principal component analysis (PCA) representing the comparison of the chemical space on active/inactive datasets in each PPI target (A, BRD; B, Cyclophilins; C, HIF1a; D, Immunophilin FKBP1A; E, Integrin; F, XIAP).


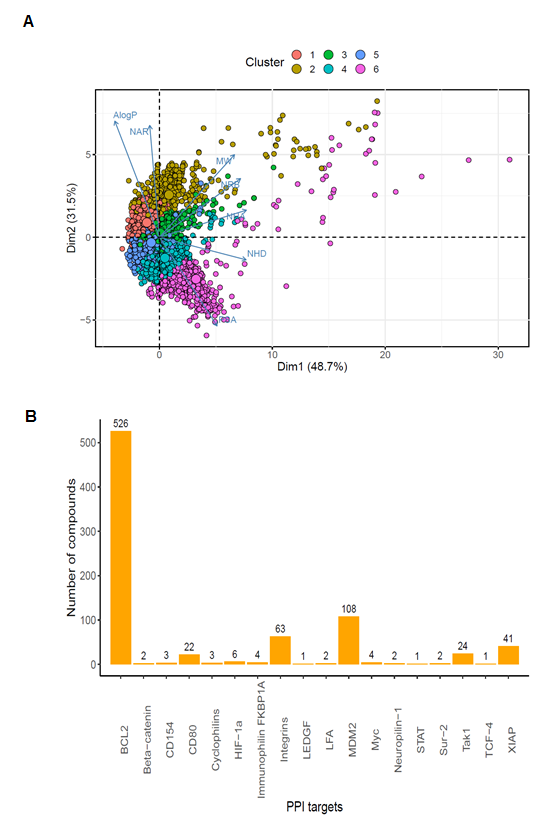


**Figure S3**. **PCA-clustering plot of the chemical space of the iPPI datasets**. (A) The loading plot vectors are represented by arrows for each physicochemical property. The red, green, blue, mustard, cyan, and magenta dots correspond to clusters 1, 2, 3, 4, 5, and 6, respectively. (B) The distribution histograms are plotted against number of targets for the compound present in the Cluster 2 dataset.


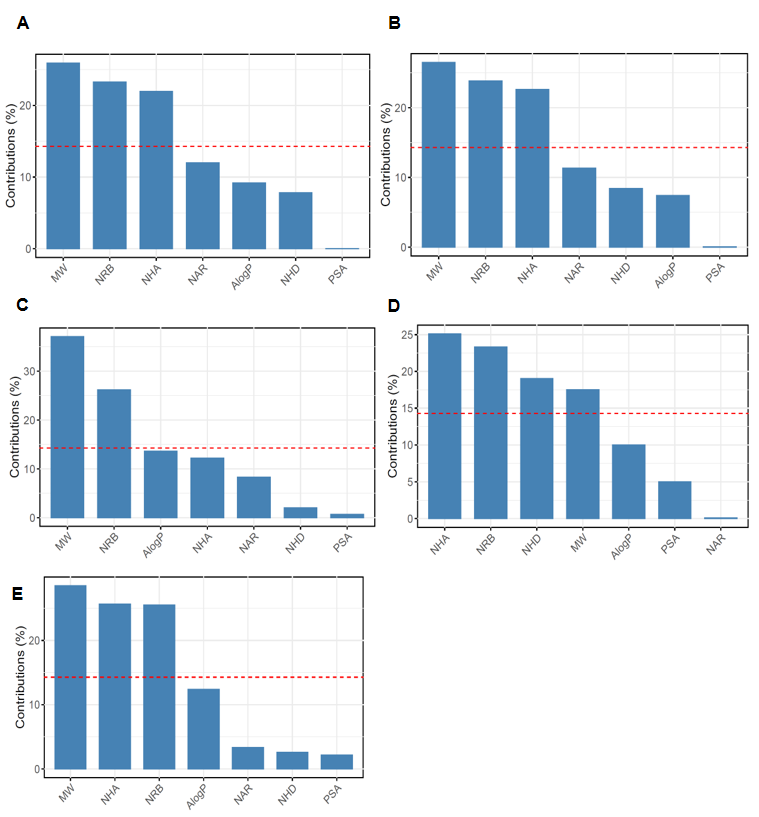


**Figure S4. Top variables contributing to the PC1 using the bar plot**. The red dashed line on the graph above indicates the expected average contribution for (A) Class 1, (B) Class 2, (C) Class 3, (D) Class 4, and (E) Class 5.

**Table S1**. Number of compounds for each PPI target used for PCA

| Target | Active compounds | Inactive  compounds |
| --- | --- | --- |
| BCL-2 | 992 | 158 |
| BRD | 194 | 20 |
| Cyclophilins | 103 | 58 |
| HIF1a | 150 | 51 |
| ImmunophilinFKBP1A | 414 | 124 |
| Integrins | 3448 | 395 |
| MDM2 | 932 | 108 |
| XIAP | 755 | 87 |
